# Using deep learning to study emotional behavior in rodent models

**DOI:** 10.3389/fnbeh.2022.1044492

**Published:** 2022-11-22

**Authors:** Jessica Y. Kuo, Alexander J. Denman, Nicholas J. Beacher, Joseph T. Glanzberg, Yan Zhang, Yun Li, Da-Ting Lin

**Affiliations:** ^1^Intramural Research Program, National Institute on Drug Abuse, National Institutes of Health, Baltimore, MD, United States; ^2^Department of Zoology and Physiology, University of Wyoming, Laramie, WY, United States

**Keywords:** deep learning, emotion, supervised learning, unsupervised learning, self-supervised learning, neural recording, pose estimation

## Abstract

Quantifying emotional aspects of animal behavior (e.g., anxiety, social interactions, reward, and stress responses) is a major focus of neuroscience research. Because manual scoring of emotion-related behaviors is time-consuming and subjective, classical methods rely on easily quantified measures such as lever pressing or time spent in different zones of an apparatus (e.g., open vs. closed arms of an elevated plus maze). Recent advancements have made it easier to extract pose information from videos, and multiple approaches for extracting nuanced information about behavioral states from pose estimation data have been proposed. These include supervised, unsupervised, and self-supervised approaches, employing a variety of different model types. Representations of behavioral states derived from these methods can be correlated with recordings of neural activity to increase the scope of connections that can be drawn between the brain and behavior. In this mini review, we will discuss how deep learning techniques can be used in behavioral experiments and how different model architectures and training paradigms influence the type of representation that can be obtained.

## Introduction

Animal behavioral studies have been used to investigate neurobiological aspects of human emotions. In this mini review, we define “emotion” as patterns of behavioral, hormonal, and autonomic responses (Dolensek et al., 2020) that explain behaviors more complex than reflexes but less complex than volitional behavior (Adolphs, 2017). The use of animal models in combination with advanced recording techniques has furthered our understanding of the biological basis of emotional behavior (Kirlic et al., 2017; Xia and Kheirbek, 2020), but precise identification of the specific neural substrates and mechanisms for emotions and emotional behavior have proved elusive (Celeghin et al., 2017).

Various brain regions have been implicated in emotional behavior, including the anterior cingulate cortex (ACC; Johnson et al., 2022), insular cortex (Dolensek et al., 2020), and subcortical deep brain structures such as the amygdala (Joëls et al., 2018; Wu et al., 2021), nucleus accumbens (NAc; Aragona and Wang, 2009), and periaqueductal gray (Buhle et al., 2013; Reis et al., 2021). Because animal models rely solely on observations of behavior to quantify emotional states, accurate and nuanced behavior quantification is required to understand the neural basis of emotional states (Xia and Kheirbek, 2020).

One approach to this problem is manual annotation of animal behavior videos by human observers, labeling behaviors of interest on a frame-by-frame basis. This process is laborious, inefficient for processing large amounts of data, error-prone, and introduces subjectivity (Arac et al., 2019; van Dam et al., 2020). The other classical approach is measuring only aspects of behavior that can easily be measured by machines, e.g., by counting lever presses in an operant chamber or using simple tracking algorithms to detect the subject’s presence in defined regions of interest (ROIs). Advancements in computer vision and deep learning have opened the door to improvement in this area. Deep learning models, inspired by the way the mammalian brain processes information, are typically trained by an iterative optimization process with large quantities of data in a fast and quasi-deterministic way (Xia et al., 2018; Richards et al., 2019; Høye et al., 2021; Contreras et al., 2022). Such models, which aim to automate the process of extracting representations from data (Arel et al., 2010; Najafabadi et al., 2015), have been applied to problems in medical imaging, natural language processing, and image recognition (Najafabadi et al., 2015; Zhao et al., 2021). Deep learning promises to allow for more nuanced analysis of emotion-related behavior than classical methods. In this mini review, we will discuss ways of measuring rodent behaviors that are relevant to anxiety, social interaction, reward, and stress responses. We will describe how deep learning can be applied to studying emotional behavior in rodent models and continues to advance our understanding of neural activity and emotional behavior.

## Paradigms for Measuring Emotional Behaviors

Researchers have studied anxiety-like behavior in rodents by using approach-avoidance conflicts. In these experiments, rewarding cues (e.g., food, drug, social interaction) elicit approach and reward-seeking behaviors (e.g., lever pressing) while aversive cues (e.g., foot shock, predatory threat) elicit avoidance and fear behaviors (e.g., freezing; Kirlic et al., 2017; Greiner et al., 2019). To assess anxiety-like behavior, researchers can present these stimuli and measure the degree to which an animal approaches or avoids them (Kirlic et al., 2017; Greiner et al., 2019). Paradigms used to measure different aspects of anxiety also include the elevated plus maze (EPM), elevated zero maze (EZM), open field test, social interaction test, hyponeophagia test, conditioned fear test, shock-probe test, Vogel conflict test, Geller-Seifter test, and the light-dark box assay (Sousa et al., 2006; Kirlic et al., 2017; Lezak et al., 2017). In conflict tests (e.g., Vogel and Geller-Seifter tests), animals seek reward (water/food) while a conflict is created by delivering punishment (shock) at a fixed ratio (e.g., every nth reward). Although deliberate avoidance is not assessed, the total shocks delivered is used as a measurement of anxiety-like behavior (Kirlic et al., 2017). Other tests measure stress responses in animals (novelty-induced suppression of feeding test, forced swim test; Sousa et al., 2006), depression-like behavior (e.g., tail suspension test; Xia and Kheirbek, 2020), or facial expression changes in reaction to different emotionally-salient stimuli (Dolensek et al., 2020).

Other classical methods focus on social behavior. Examples include the social interaction test, social preference-avoidance test, social approach-avoidance test, three-chambered social approach test, modified Y-maze, and tests that observe maternal behavior (Sousa et al., 2006; Kirlic et al., 2017). In the social interaction test, social and exploratory behaviors of two rodents are monitored in a familiar or unfamiliar context with different lighting conditions. The amount of time rodents spend interacting under the four test conditions is used as a measure of anxiety-like behavior. Other commonly quantified emotional behaviors include freezing, defecating, vocalizations, and self-grooming (File and Seth, 2003; Sousa et al., 2006). Conditioned place preference paradigms have been used to investigate social vs. drug reward in rodents where time spent in social or drug chambers are recorded (Thiel et al., 2008; Kummer et al., 2014). An operant social self-administration protocol described by Venniro and Shaham (2020) demonstrated volitional operant choice of social over drug reward where total lever presses for each reward was used as a measure of operant social choice.

Measurements such as rate, time, and number of task-related behaviors performed are convenient ways to quantify observable emotional behavior. However, many current assays rely on rudimentary measurements and generalized assumptions that reduce translational value. For example, we assume that animals in the EPM that spend less time in the open arms are more anxious than others that spend more time (Lezak et al., 2017). Measures like lever press counts and time spent in certain zones are not fully representative of complex internal emotional states. Animal movement and posture captured on video can convey much more information about behavior and internal state, but accessing that information in a systematic way is a major challenge in image processing and machine learning. Recent advances in pose estimation and emerging methods for behavioral analysis hold the potential to vastly increase the richness and variety of behavioral variables available for analysis.

## Tracking and Analyzing Pose Estimation Data

The modern deep learning revolution began in 2012 when a convolutional neural network (CNN) reached human-level accuracy visual recognition (Serre, 2019; Mathis and Mathis, 2020). Since then, many techniques have been developed to apply deep learning algorithms to animal behavior analysis, including pose estimation algorithms [e.g., DeepLabCut (DLC), SLEAP; Mathis et al., 2018; Pereira et al., 2022]. Nath et al. (2019) highlights the DLC protocol, in which users manually label body parts in a subset of frames and use those labels to train a pose estimation model. The trained model is used to extract information about the subject’s pose, or geometric configuration of body parts (Mathis et al., 2018), from a large corpus of behavioral video data. DLC has been developed to accommodate more experimental setups, including top-down, bottom-up, and multi-animal 3D tracking (Lauer et al., 2022).

The output of pose estimation workflows (such as DLC) is frame-by-frame information about the location of the labeled body parts (if detected) within the video frame. For research focused primarily on tracking kinematics or an animal’s location (e.g., presence within some ROI), this may be sufficient for the desired analyses. For example, DLC has been used in EPM, EZM, and open field tests to measure time spent in different areas, distance traveled, location, and velocity (Cui et al., 2021; Lu et al., 2021; Sun et al., 2021; Johnson et al., 2022; Sánchez-Bellot et al., 2022). However, pose estimation also offers the possibility of extracting more general information about an animal’s actions and behavioral state from moment to moment. This area of study has attracted significant attention in recent years. A number of methods have been proposed for extracting this type of information from video data, a comparison of which is the main focus of this review.

## Machine Learning Approaches for Emotional Behavioral Analysis

A variety of frameworks for deriving behavioral motifs and classifying behavioral states using pose estimation or related methods have been proposed (Table 1). Supervised approaches use pose data to identify experimenter-specified behaviors. Unsupervised approaches seek to identify naturally occurring behavior categories by clustering. Self-supervised approaches involve training a model to predict some variable derived from the data itself (rather than directly set by the experimenter) and the trained model is used as a feature extractor for subsequent clustering or other analyses. These three learning types affect the type of behaviors that may be identified.

**Table 1:** Behavior classification algorithms.

	Supervised	Self-Supervised	Unsupervised
Uses tracking data	SimBA, JAABA, DLC Analyzer	DBM, VAME, Selfee	B-SOiD, PyRAT
Built-in pose estimation	MARS	-	AlphaTracker
Does not use tracking data	-	BehaveNet	MoSeq, MotionMapper

**Figure 1:**
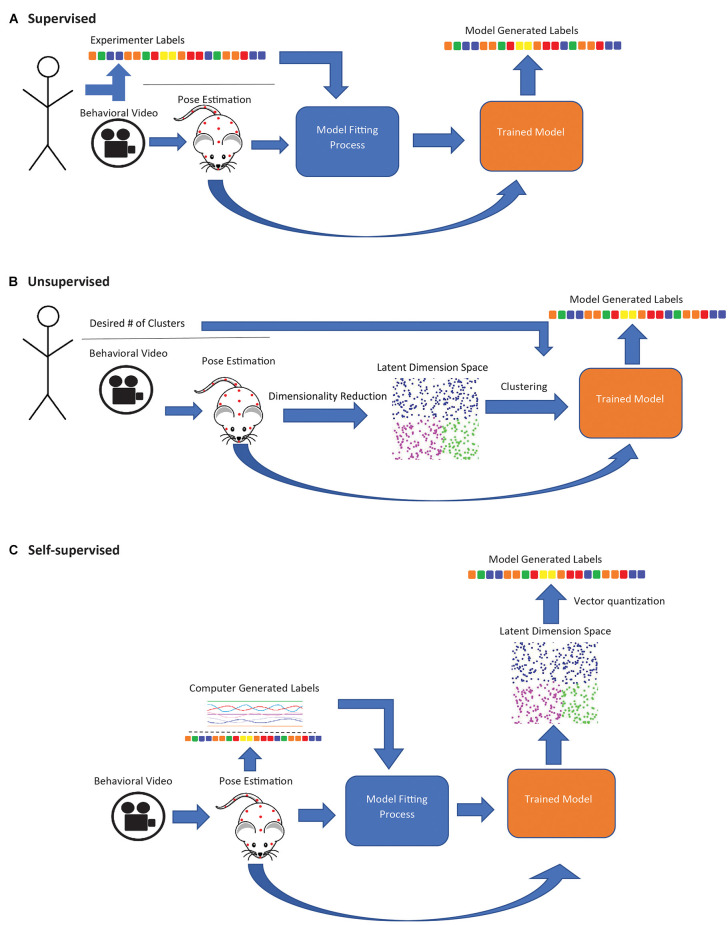
Schematic diagram of supervised, unsupervised, and self-supervised approaches for animal behavior classification. **(A)** In supervised approaches, a model is trained to generate behavioral classifiers that replicate human annotations. **(B)** Unsupervised approaches are entirely data driven; pose estimation data is compressed to the latent state representation and clustered to maximize similarities in data points. Experimenters may sometimes specify the number of desired clusters. **(C)** In self-supervised approaches, a model is trained to generate labels derived from the data. The trained model is used to map subject behavior to a latent space, and the space is then discretized (usually with K-means).

Supervised approaches employ a classification model trained on human-annotated data, which is then applied to new data (Figure 1A). SimBA (Nilsson et al., 2020) uses features extracted from DLC or SLEAP pose data as input to a random forest classifier to label mouse behaviors, such as sniffing (social behavior; Dawson et al., 2022), freezing (fear conditioning; Hon et al., 2022), and pup retrieval (Winters et al., 2022). DLCAnalyzer (Sturman et al., 2020) also includes capabilities for supervised behavior classification and has been used to track head dips in the EPM (Grimm et al., 2021) and head angle in the open-field test (von Ziegler et al., 2022). MARS combines pose estimation and behavior classification capabilities and is suitable for multi-animal social behavior analysis (e.g., attack, mounting; Segalin et al., 2021). These approaches are most suitable when researchers are interested in specific behaviors known *a priori*, as opposed to unsupervised or self-supervised approaches which offer no guarantee that any specific behavior will be identified as a distinct category. However, human annotations can be biased, subjective, and may not capture subtle differences in how a behavior is performed (Pereira et al., 2020). The utility of supervised approaches is limited in situations where researchers wish to uncover naturally occurring behavioral motifs. Additionally, the above frameworks all attempt to classify behavior within a narrow temporal window (e.g., features extracted in the SimBA workflow are calculated over a rolling 500 ms window; Nilsson et al., 2020), limiting their ability to identify highly contextual behavioral motifs or larger-scale patterns of behavior.

Unsupervised learning models, in contrast to supervised models, are entirely data driven and require minimal experimenter input (Figure 1B). These models generate behavioral clusters based on similarities and differences between data points, e.g., video frames (Goodwin et al., 2022). Unsupervised methods generally involve a dimensionality reduction step, where a large set of input features are compressed into a low-dimensional representation, and a clustering step, where data points (i.e., frames in a video sequence) are clustered to maximize the similarity of points within a cluster, which in some cases requires the experimenter to specify the number of clusters. B-SOiD (Hsu and Yttri, 2021) and PyRAT (De Almeida et al., 2022) apply nonlinear dimensionality reduction, which can capture nonlinear relationships in the input data (Portnova-Fahreeva et al., 2020), while AlphaTracker (Chen et al., 2020) uses linear dimensionality reduction (principal component analysis, PCA). All three methods then group video frames into behavioral categories through hierarchical clustering. MoSeq (Wiltschko et al., 2015) takes a different approach, applying linear dimensionality reduction directly to video frames (without pose estimation) and segmenting the resulting continuous representation of behavior using an autoregressive hidden Markov model (ARHMM; Bryan and Levinson, 2015), a variant of the hidden Markov model adapted to modeling continuous valued time series as the product of an underlying sequence of discrete states, making it potentially better suited than simple clustering-based approaches to identify temporal patterns over a longer timescale.

Using unsupervised methods is advantageous when the experimenter wishes to label behaviors without choosing specific behaviors, or to uncover variations in how behaviors are performed. Approaches that use hierarchical (PyRAT, AlphaTracker) or density-based clustering methods (B-SOiD) do not even require that the user specify the number of behavioral clusters. This property is a double-edged sword, however, as hierarchical and density clustering algorithms do not attempt to ensure that all points included within a cluster are similar to each other, only that there is no clear dividing line. Thus, they are suited to detecting behaviors that occur in sustained bouts with relatively brief transitions between behaviors, but may group very different behaviors together when transitions between behaviors occur very frequently.

Self-supervised methods (Figure 1C) combine aspects of both supervised and unsupervised learning. Like supervised methods, they rely on classification or regression models (usually a deep neural network), but the model is trained to produce outputs derived from the data instead of manual annotations (in the simplest case, the autoencoder, the output is the same as the input). After training, the final classification/regression output layers of the model are discarded, and the model is used as a feature extractor (Misra and van der Maaten, 2019). Applied this way, the model performs nonlinear dimensionality reduction, mapping video frames to points in a feature space, with the choice of method for deriving the training outputs strongly influencing which aspects of the pose/video data are represented in that space. This “mapping” step may be followed by a discretization step, in which the transformed points (i.e., frames) are grouped into categories (typically using a vector quantization algorithm such as K-means). These categories are usually assigned descriptive labels by visually inspecting video clips to determine what behavior(s) each category is associated with.

Deep behavior mapping (DBM; Zhang et al., 2022) is a self-supervised framework, in which training labels are derived by assigning different labels to video frames according to which ROI the subject is in, in combination with time windows around experimental events (e.g., lever press). DBM, which uses pose data from DLC as input and employs a long-short term memory (LSTM; Hochreiter and Schmidhuber, 1997) classification network and discretizes the extracted feature space using K-means, has been used to capture behavioral microstates in a mouse operant task, including identifying distinct phases of a well-learned behavior sequence (e.g., lever approach, lever interaction, shifting from lever port to food port, food port search, food consumption) and also various non-task behaviors (grooming, rearing, visiting water sipper; Zhang et al., 2022). VAME (Luxem et al., 2022) uses a similar model architecture (using gated recurrent units (Cho et al., 2014), a variant of LSTM), but trains the model using only the pose data itself. The model is trained to reproduce the input pose sequence (i.e., an autoencoder), plus predict the pose data a short distance into the future. This approach has the benefit of not requiring the experimenter to specify rules for deriving output labels for training, at the cost of potentially making the process more sensitive to noise and occlusions (Hausmann et al., 2021; Luxem et al., 2022). Selfee (Jia et al., 2022) and BehaveNet (Batty et al., 2019), unlike VAME and DBM, operate directly on snippets of video rather than pose data from DLC/SLEAP, employing autoencoder-style training directly on video data for nonlinear dimensionality reduction. Selfee operates on short 3-frame snippets of raw video, has been used in mice to identify behaviors such as social nose contact and allogrooming in open field tests, and places its main emphasis on using the extracted feature space for a variety of downstream analyses (Jia et al., 2022). BehaveNet, unlike the other self-supervised methods, performs feature extraction on individual frames rather than sequences of frames, and does not consider the temporal structure of the data until the discretization step, which uses an autoregressive hidden Markov model (Batty et al., 2019).

Data with occluded views of experimental subjects present an additional challenge for pose estimation-based methods (Mathis and Mathis, 2020); for example, recording animals top-down can hide the feet of an animal, while top-down recordings of animals with headcaps or microendoscopes often suffer intermittent occlusion of parts of the head. Recording multiple animals interacting can also occlude body parts, but advances in 3D animal tracking (Marshall et al., 2022) can help minimize the effect of occlusions on tracking of animal identity. Another challenge with recording multiple animals is that pose-estimating algorithms like DLC may not be able to differentiate between similar looking animals (Lauer et al., 2022). AlphaTracker (Chen et al., 2020) does both tracking and behavior classification and could reliably track four identical-looking mice. The quality of the available data is likely to be a major determining factor in which types of methods are applicable to a problem, with unsupervised (e.g., B-SOiD, PyRAT) or autoencoder-based methods (VAME, BehaveNet, Selfee) best suited to handling relatively noise-free data, and classifier-based methods (e.g., SimBA, DBM) better suited to handling data with frequent occlusions and lower video quality.

## Correlating Neural Activity with Emotional Behavior

Advances in imaging technology such as 1-photon miniscope calcium imaging allow for *in vivo* imaging of neuronal activity in freely behaving animals. Using techniques that record from freely moving animals allows for studying an animal’s neuronal activity in more complex behavioral paradigms (Laing et al., 2021). Recent miniscope imaging studies in freely moving animals correlate calcium activity with complex behaviors such as pain behavior (Liu et al., 2022), defensive behavior (Kennedy et al., 2020; Ponserre et al., 2022), and reward behavior (Siemian et al., 2021).

Many correlations between neural activity and animal behavior are based on measurements such as timestamps. Integrating neural imaging and machine/deep learning techniques in rodents allows for more nuanced relationships to be identified. Studies have utilized machine/deep learning techniques for head-fixed *in vivo* 2-photon calcium imaging in mice (Dolensek et al., 2020; Lu et al., 2021; Yue et al., 2021). However, many aspects of emotional behavior are best measured when rodents are freely behaving in the testing area (e.g., grooming, nesting, or approach/avoidance). Recent studies have utilized miniscope calcium imaging to allow recording of neural activity in freely moving animals, which can then be combined with advanced techniques for behavior analysis (Zhang et al., 2022). DLC-derived data in a rat exposure assay was used for automated classification of defensive behaviors and correlated with dorsal periaqueductal gray activity (Reis et al., 2021). DLC-derived data from mice in the EZM and open field tests was used to extract kinematics and identify correlations between the activity of interneurons in the ACC and anxiety and social behavior (Johnson et al., 2022). In a study on prosocial affiliative touch, a deep CNN and an LSTM-based RNN identified that affiliative allogrooming was controlled by the medial amygdala (Wu et al., 2021).

*In vivo* electrophysiology can also be combined with video recording in freely moving animals. For example, the firing rate of NAc and dorsolateral striatum neurons changed with operant vertical head movement as drug intake increased (Coffey et al., 2015). Pose estimation and deep learning-based behavioral analysis can also be used with optogenetic manipulation to understand functions of neuron ensembles in behavior (Grieco et al., 2021). For example, optogenetic stimulation of primary motor cortex in marmosets used DLC tracking of hand positions to identify neurons involved in forelimb movement (Ebina et al., 2019). Integrating deep learning analysis and neural recording in behavioral studies allows for correlation of neurons with behaviors in greater detail.

## Discussion

The advent of machine learning and deep learning enables the simultaneous specialization and standardization of behavior classification processes across neuroscience research. Previous studies have utilized various deep learning techniques to look at behaviors that correlate with pain states (Bohic et al., 2021), social behavior in stressed mice (Rodriguez et al., 2020; Lee et al., 2022), depressive behavior in stressed mice (Rivet-Noor et al., 2022), and reward behavior in mice models of food seeking (Zhang et al., 2022) or drug and alcohol abuse (Campos-Ordoñez et al., 2022; Neira et al., 2022). This allows behavioral neuroscientists to explore behavioral nuances in a more generalizable way (Goodwin et al., 2022). As deep learning algorithms continue to evolve, we predict more integration of deep learning and neural recording techniques to elicit the neural mechanisms of behavioral motifs implicated in emotion.

Deep learning techniques can help parse connections between behavior and the brain in complicated situations. For example, various cell types, circuits, systems, and projections in the brain have roles in multiple emotional behaviors, evidenced by chronic pain and depression comorbidity (Taylor et al., 2015) or addiction and anxiety comorbidity (Greiner et al., 2019). There are many complex interactions and overlaps between neural activity that underlie emotional behaviors, and much remains to be discovered. Another example is that the mu-opioid system has been implicated not only in pain and reward, but also in modulation of social-emotional behaviors (Meier et al., 2021). Additionally, salience and affect play important roles in pain, as affective systems, motivational systems, and pain processing interact (Taylor et al., 2015). Combining neural recording techniques with behavior analyses that allow fine-grained differentiation of behavioral states and motifs can help resolve such paradoxes by providing a broader range of hypotheses to explain correlations between neural activity and behavior. This allows researchers to refine our understanding of the precise role each neural circuit plays in the larger interaction of internal state, external stimulus, and behavior. In this way, deep learning techniques advance our understanding of the neuronal mechanisms of emotional behavior in animal models, and the importance of using these methods has strong translational power to treat mood disorders, addiction, pain, and other emotional disorders in humans.

## Author Contributions

JK wrote the first draft of the manuscript. AD developed DBM. All authors contributed to the article and approved the submitted version.

## Funding

This research was supported by NIH NIDA IRP. JK is supported by the NIDA IRP Scientific Director’s Fellowship for Diversity in Research. NB and YZ is supported by the Post-doctoral Fellowship from the Center on Compulsive Behaviors, National Institutes of Health. YL is supported by National Institutes of Health grants 3P20GM121310-05S1, R61NS115161, and 4UH3NS115608-02.

## Conflict of Interest

The authors declare that the research was conducted in the absence of any commercial or financial relationships that could be construed as a potential conflict of interest.

## Publisher’s Note

All claims expressed in this article are solely those of the authors and do not necessarily represent those of their affiliated organizations, or those of the publisher, the editors and the reviewers. Any product that may be evaluated in this article, or claim that may be made by its manufacturer, is not guaranteed or endorsed by the publisher.
